# Case Report: Intraosseous schwannoma of the thoracic spine: two case reports and an updated review of the literature

**DOI:** 10.3389/fonc.2025.1651417

**Published:** 2025-10-22

**Authors:** Hangqi Hu, Dan Cao, Houyun Xu, Xiping Yu, Xian Wang, Jibo Hu

**Affiliations:** ^1^ Department of Medical Oncology, Sir Run Run Shaw Hospital, Zhejiang University School of Medicine, Hangzhou, China; ^2^ Department of Radiology, Sir Run Run Shaw Hospital, Zhejiang University School of Medicine, Hangzhou, China; ^3^ Department of Radiology, The Fourth Affiliated Hospital, Zhejiang University School of Medicine, Yiwu, China; ^4^ Department of Pathology, The Fourth Affiliated Hospital, Zhejiang University School of Medicine, Yiwu, China

**Keywords:** intraosseous schwannoma, spinal, thoracic vertebra, surgery, case report

## Abstract

Intraosseous schwannomas (IOSs) are benign tumors composed of nerve sheath cells, representing less than 0.2% of primary bone tumors. These tumors most commonly affect the mandible and sacrum, while vertebral involvement remains exceedingly rare. Herein, we present two cases of SISs located at the T2 and T8 vertebrae. We detail the clinical presentation, imaging features, histopathological characteristics, and surgical management of SISs to advance the understanding of this rare condition. Typically presenting as expansile lytic lesions with sclerotic margins, lacking periosteal reaction or calcification, SISs frequently cause foraminal widening and vertebral scalloping, which are characteristic imaging findings that differentiate them from other bone tumors. Complete tumor resection is the recommended treatment, and spinal fusion is often necessary for reconstruction, with recurrence being uncommon after surgery. Additionally, we conduct a review of the existing literature and engage in a discussion regarding this uncommon tumor, enriching clinicians’ differential diagnosis of vertebral body osteolytic lesions and providing valuable clinical experience for the individualized selection of surgical approaches.

## Introduction

Spinal schwannomas are benign tumors that arise from nerve sheath cells, typically located in the intradural extramedullary region of the spinal cord, and they seldom invade the bone (1). When these tumors exhibit invasive traits and cause osteolytic destruction of the bone, they are categorized as intraosseous schwannomas (IOSs).

IOSs are extremely rare, accounting for less than 0.2% of primary bone tumors, and spinal intraosseous schwannomas (SISs) represent an even more uncommon subset (2). To date, there is no widely recognized definition for SIS. In our study, we define SIS as a tumor that induces invasive and osteolytic destruction of the spine, irrespective of the size of the extraosseous portion (3). The PUTH classification is an anatomical classification system developed for cervical dumbbell tumors, covering all tumor types and offering practical guidance for surgical approaches (4). Consequently, we utilized the PUTH classification to categorize SISs and inform the selection of surgical approaches, with SISs classified under Section V.

The first case of SIS was reported in 1971 by Dickson et al. (3). A thorough review of the literature indicates that only 9 cases of SISs found in the thoracic vertebrae have been reported (**Table 1**). Herein, we report two cases of SISs located at the T2 and T8 vertebrae. We detail the clinical presentation, imaging features, histopathological characteristics, and surgical management of SISs to advance the understanding of this rare condition. Furthermore, we conduct a review of the existing literature and engage in a discussion regarding this uncommon tumor, enriching clinicians’ differential diagnosis of vertebral body osteolytic lesions and providing valuable clinical experience for the individualized selection of surgical approaches.

**Table 1:** Summary of the previously reported cases and two new cases of intraosseous thoracic schwannomas.

No.	Year/Author	Age/Sex	Level	Approach	Stabilization	Pathology	Resection	Prognosis	Symptoms
1	1997/ Nooraie (18)	46/M	T12	Posterior	Fusion + fixation	Benign	Complete	1.5 years no recurrence	Burst fracture caused by trauma
2	2001/ Ramasamy (1)	37/M	T12	Anterior + posterior	Fusion + Fixation	Schwannoma with proliferation	Complete	1.5 years no recurrence	Back pain, weakness and numbness of lower limbs
3	2007/ Choudry (19)	18/M	T12	Anterior	Fusion + fixation	Benign	Complete	5 years no recurrence	Back pain, weakness of both lower limbs
4	2009/ Cetinkal (20)	55/F	T12	Posterior	No	Benign	Complete	1 year no recurrence	Back pain, pain and numbness of right lower limb
5	2010/ Kojima (21)	60/M	T9	Posterior	Pedicle screw fixation	Benign	Complete	2 years no recurrence	Back pain, weakness and numbness of lower limbs
6	2015/ Zhang (22)	54/F	T9	Posterior	Fusion + fixation	Benign	Complete	4 years no recurrence	Paresthesia and numbness of both lower limbs
7	2018/ Jia (23)	64/F	T7-T8	Posterior	Fusion + fixation	Benign	Complete	1 year no recurrence	Back pain, weakness
8	2019/ Zaidman (24)	56/F	T1	Anterior	Fusion + fixation	Benign	Complete	Not mentioned	Asymptomatic
9	2024/ Rahyussalim (6)	40/M	T9	Posterior	Vertebroplasty	Benign	No	1 year symptoms improvement	Back pain, numbness and tingling sensation of left lower limb
10	2025/ Present case	31/F	T2	Anterior + posterior	Fusion + fixation	Benign	Complete	2 years no recurrence	Back pain and numbness of both lower limbs
11	2025/ Present case	58/F	T8	Anterior	Fusion + fixation	Benign	Complete	1 year no recurrence	Asymptomatic

F, female; M, male.

## Case description

### Case 1

A 31-year-old female presented with a two-month history of mild numbness in both lower limbs, particularly in the thighs. Over the past ten days, the numbness gradually intensified, accompanied by low back pain and unsteadiness while walking. Upon admission, a physical examination revealed diminished sensation in the lower extremities; however, muscle strength remained normal.

**Figure 1:**
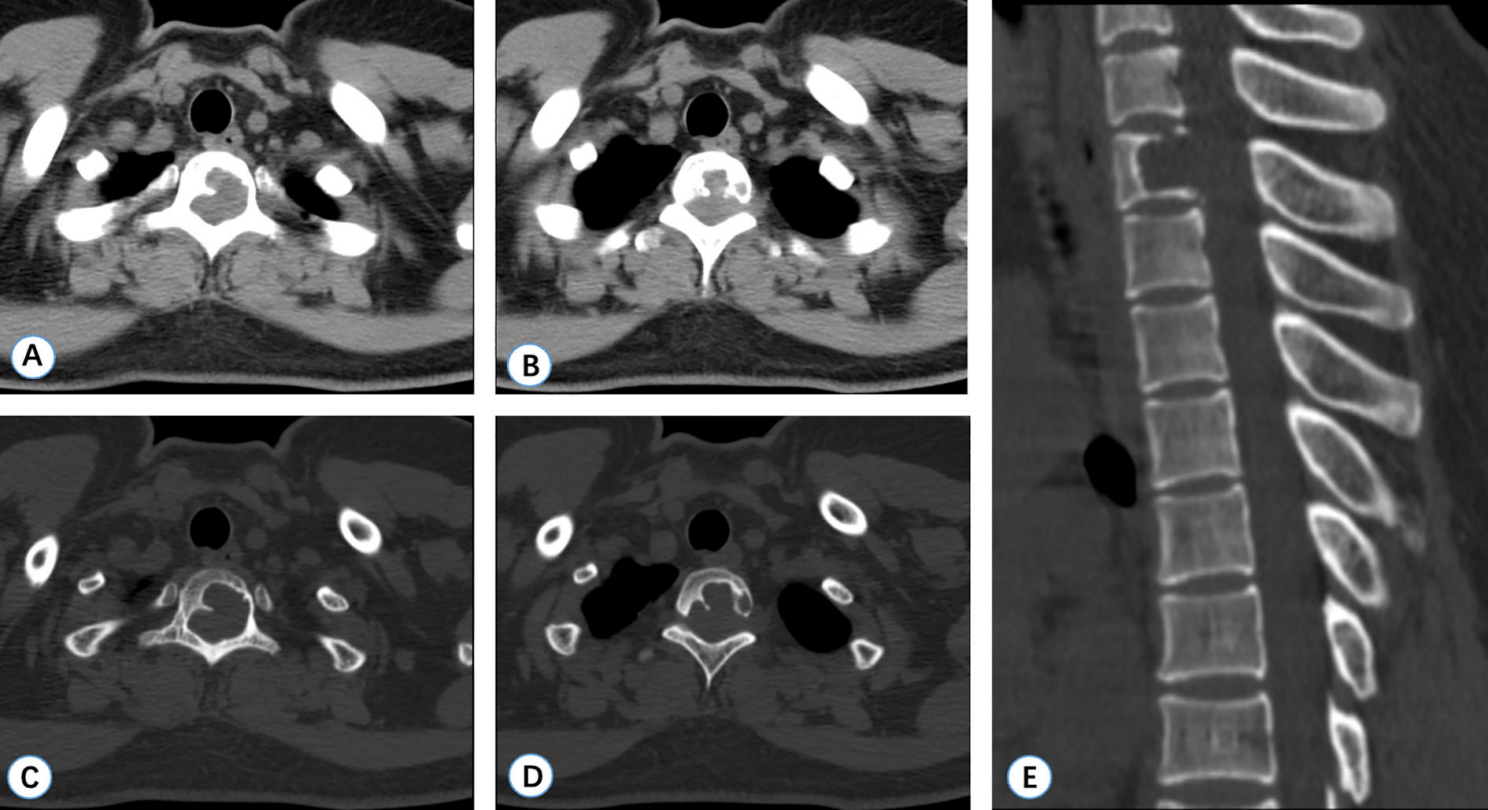
CT manifestations of Case 1 (T2 vertebra). **(A–D)** Axial CT scans reveal osteolytic bony destruction of the T2 vertebra with intraspinal extension. **(E)** A sagittal CT scan demonstrates a bony defect in the T2 body.

**Figure 2:**
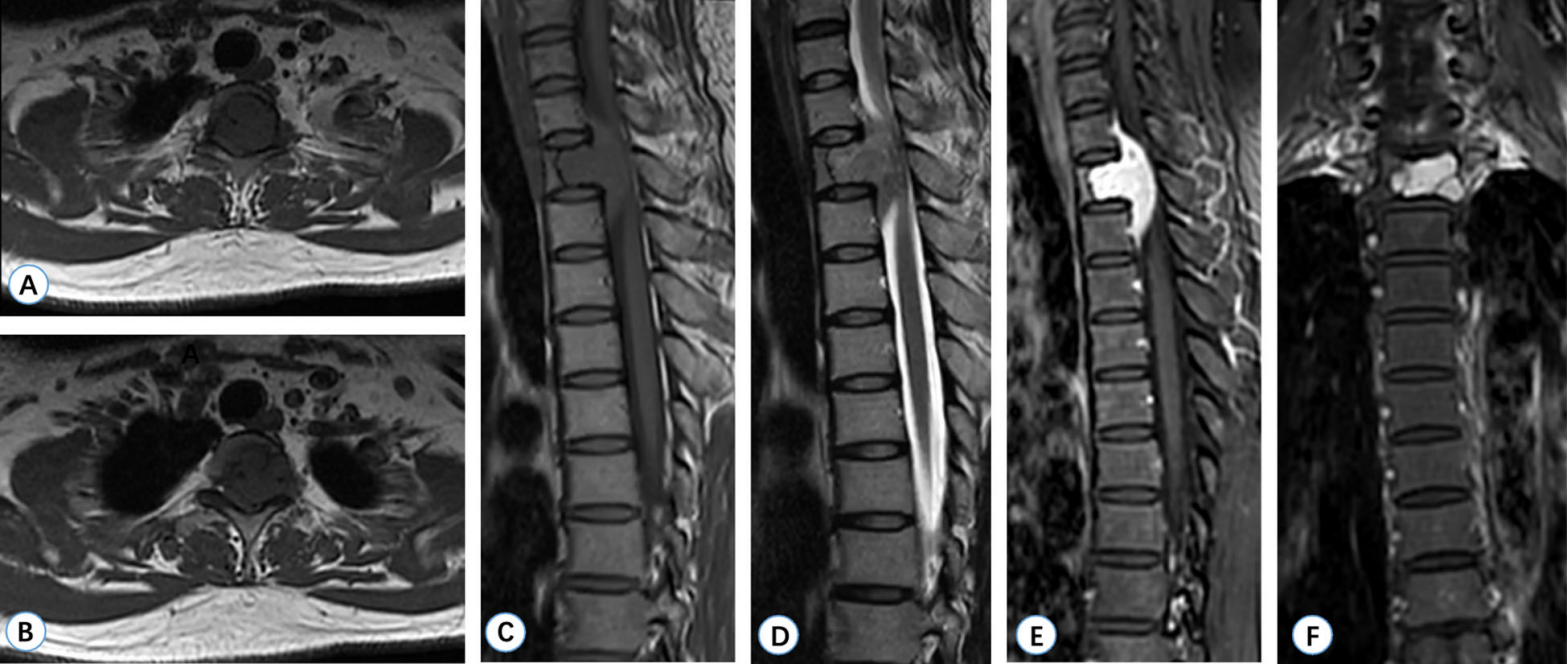
MRI manifestations of Case 1 (T2 vertebra). **(A, B)** Axial T1-weighted images reveal a lobulated, well-defined hypointense mass. **(C)** The sagittal T1-weighted image demonstrates that the tumor is isointense relative to the spinal cord. **(D)** The sagittal T2-weighted image indicates that the tumor exhibits high mixed intensity, accompanied by spinal cord compression. **(E, F)** Gadolinium-enhanced T1-weighted imaging displays significant irregular enhancement.

**Figure 3:**
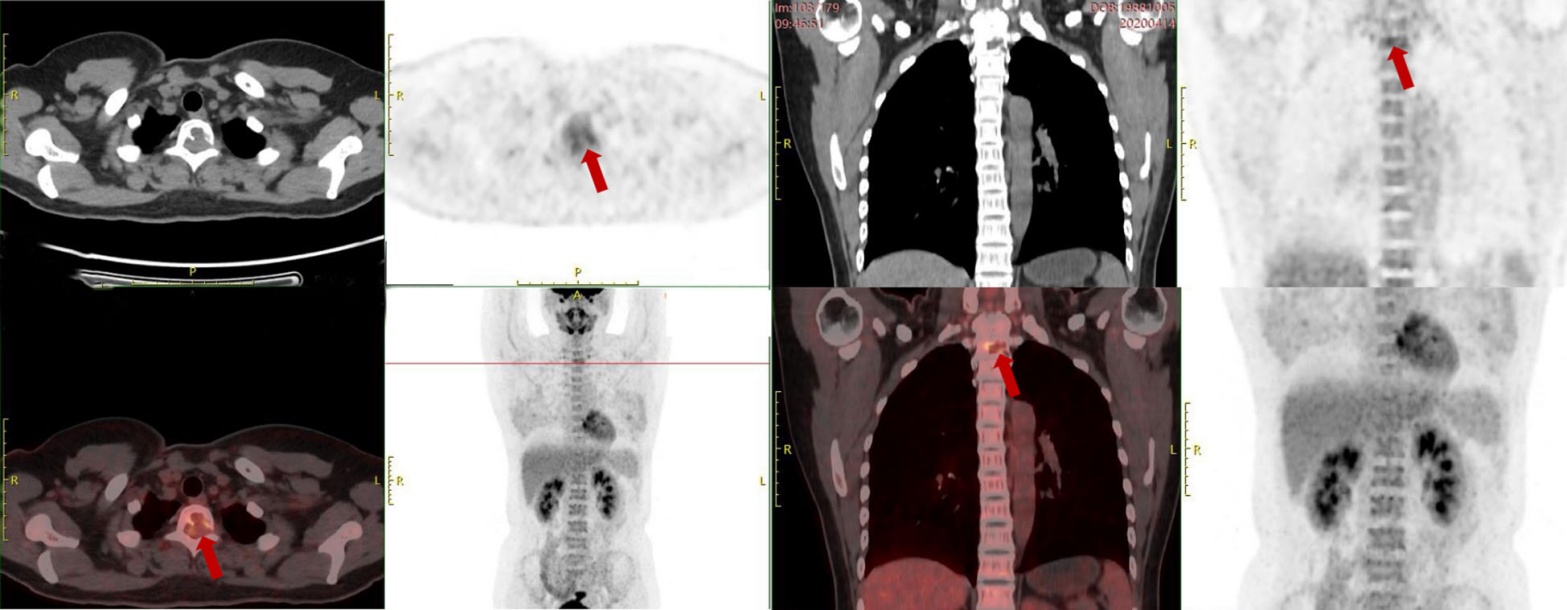
^18^F-fluorodeoxyglucose positron-emission tomography-computed tomography (^18^F-FDG-PET/CT) reveals a hypermetabolic lesion in the T2 vertebral body (SUX max=4.2) (indicated by the red arrow).

Computed tomography (CT) revealed an irregular lytic lesion with marginal sclerosis located at the T2 vertebra, measuring 21 mm *×* 13 mm. The lesion protruded into the spinal canal, compressing the dural sac and involving the left pedicle (**Figure 1**). Magnetic resonance imaging (MRI) of the thoracic spine demonstrated a lobulated, well-circumscribed mass that exhibited heterogeneous hyperintensity on T2-weighted imaging (T2WI) and iso-intensity on T1-weighted imaging (T1WI). Significant homogeneous enhancement was observed following gadolinium-DTPA injection. The lesion extended into the spinal canal, resulting in compression and displacement of the spinal cord at the corresponding level (**Figure 2**). ^18^F-fluorodeoxyglucose positron-emission tomography-computed tomography(^18^F-FDG-PET/CT) revealed positive FDG uptake in the T2 vertebra lesion (SUV max = 4.2), while other areas showed no abnormal FDG uptake (**Figure 3**).

The patient underwent resection of the lesion using a combined anterior and posterior approach. The tumor was well-defined and extended into the spinal canal, but it did not involve the nerves or spinal cord, nor were there any dural adhesions. Following the removal of the residual lamina and spinous process, the tumor was completely excised. Transpedicular screws and intervertebral bone fusion were then applied to stabilize the spine. Transfemoral spinal angiography indicated that the lesion had a rich blood supply; consequently, preoperative embolization was performed to minimize intraoperative bleeding.

**Figure 4:**
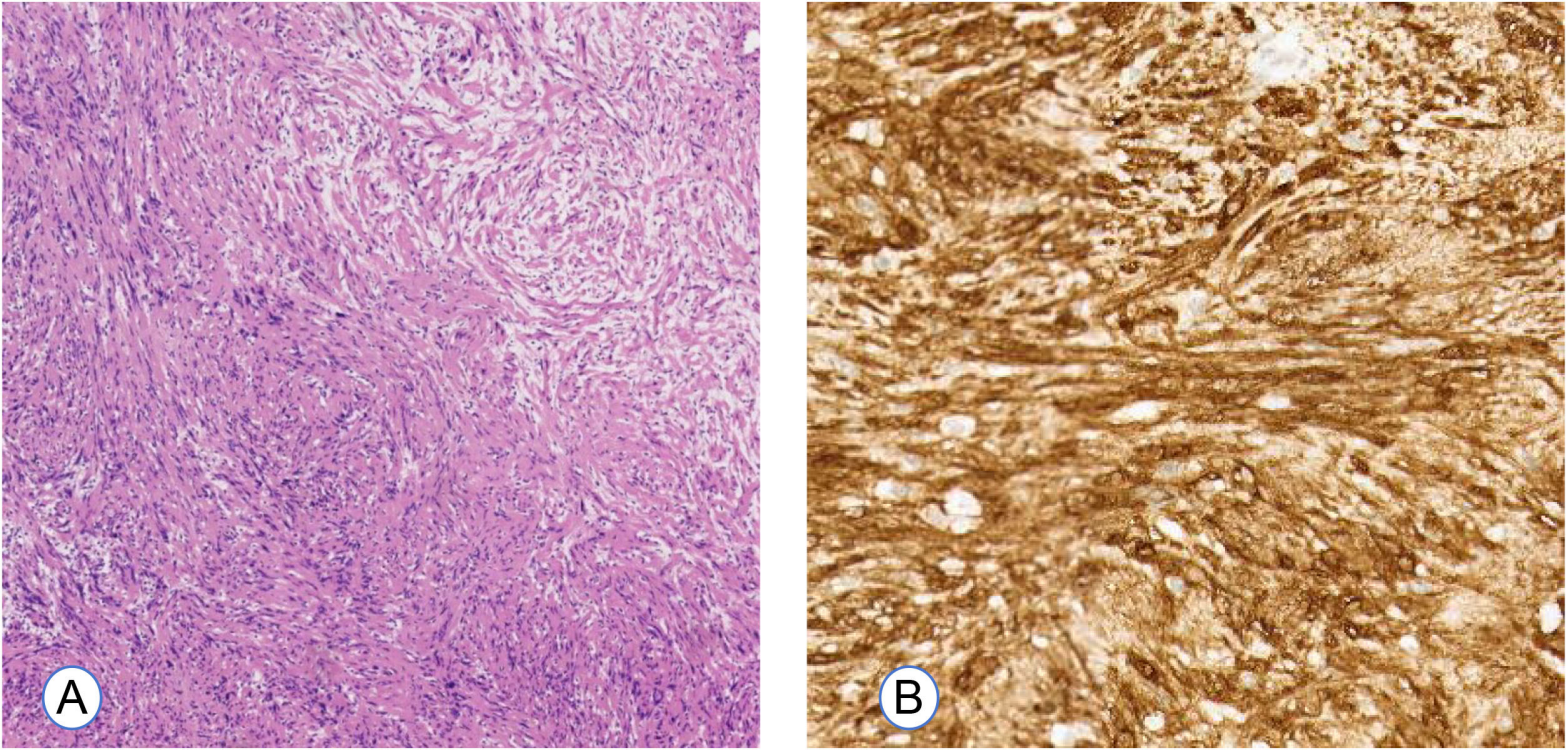
Histopathological examination of Case 1. **(A)** Histological analysis shows hypercellular (Antoni A) and hypocellular (Antoni B) regions, consistent with a typical schwannoma. (H&E, original magnification ×4). **(B)** Immunohistochemistry showing S-100 protein over-expression (brown color, ×10).

Microscopic examination revealed compact cellular regions characterized by spindle-shaped cells exhibiting palisading of nuclei (Antoni A), interspersed with areas of less cellular myxoid connective tissue (Antoni B). Immunohistochemical analysis revealed S-100 positivity, CD34 negativity, and a Ki-67 index of 2-3% (**Figure 4**). The pathological features of the tumor were consistent with a diagnosis of schwannoma.

**Figure 5:**
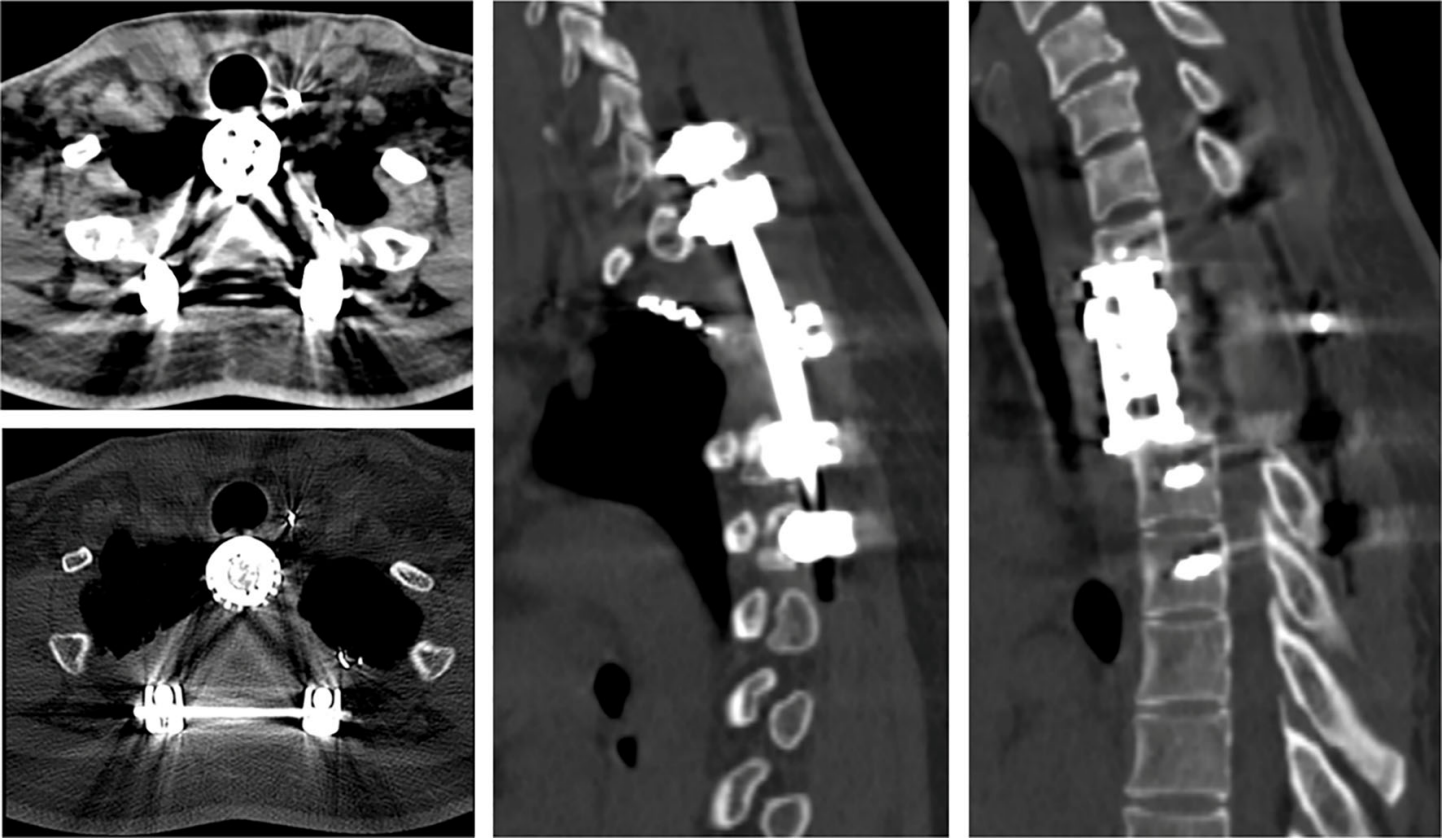
CT imaging reveals no evidence of recurrence throughout the 2-year follow-up period, with internal fixation in place.

Postoperatively, the patient experienced an uneventful recovery, demonstrating no neurological impairment. Throughout the 2-year follow-up period, the patient remained asymptomatic. CT imaging revealed no evidence of recurrence (**Figure 5**).

### Case 2

A 58-year-old female patient was admitted to our department after being diagnosed with a mediastinal tumor during a routine physical examination. She had a medical history of hypertension and lumbar disc herniation.

**Figure 6:**
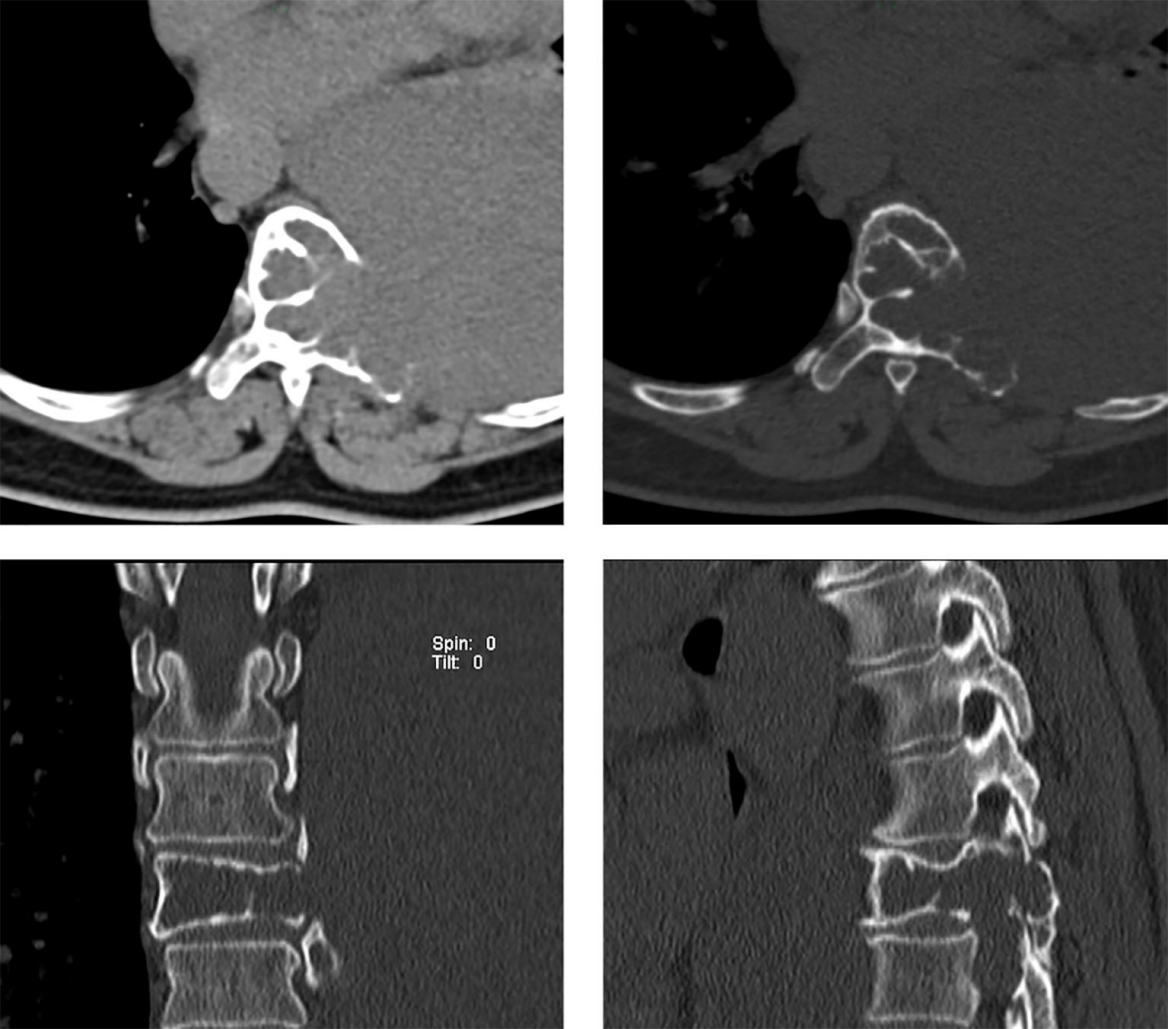
CT manifestations of Case 2 (T8 vertebra). Axial CT scan reveals bone destruction in the T8 vertebrae and the left costotransverse joint, and irregular soft tissue density shadows were observed in the T8 vertebra, which protruded into the thoracic cavity on the left side.

**Figure 7:**
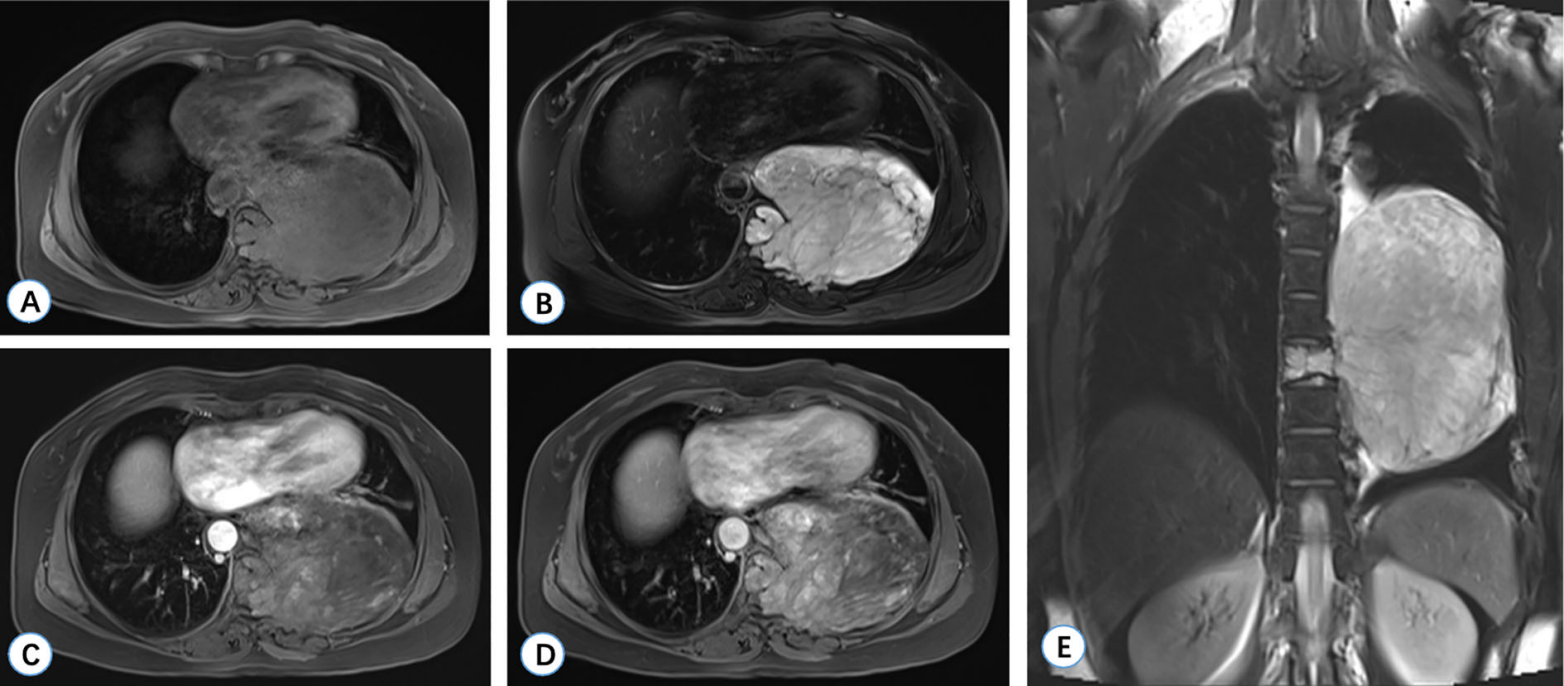
MRI manifestations of Case 2 (T8 vertebra). **(A)** An axial T1-weighted image reveals a large lobulated hypointense mass at the T8 vertebra, protruding into the thoracic cavity on the left side. **(B)** An axial T2-weighted image demonstrates heterogeneous hyperintensity of the tumor. **(C, D)** Enhanced T1-weighted images indicate mild irregular enhancement. **(E)** A coronal T2-weighted image shows that the tumor originates from the T8 vertebral body.

Axial CT revealed a large, well-circumscribed mass in the left thoracic cavity, measuring 95 mm × 126 mm × 131 mm, with homogeneous density. This mass involved the partial thoracic vertebrae (T8) and the 7th to 9th costotransverse joints (**Figure 6**). Preoperative MRI demonstrated an osteolytic expansile mass at T8, appearing hypointense on T1WI and heterogeneously hyperintense on T2WI. This mass was connected to the soft tissue mass in the thoracic cavity via the intervertebral foramen and spinal canal. Notably, the spinal cord was compressed and displaced at the corresponding level, although there was no significant involvement observed. On contrast-enhanced T1WI, the tumors exhibited mild heterogeneous enhancement (**Figure 7**).

A large dumbbell-shaped tumor was identified in the chest cavity following surgery through the bed of the 7th rib. The tumor was well-encapsulated and highly vascularized. It extended into the spinal canal via the intervertebral foramen and was connected to the intrathoracic mass at the 8th vertebra, spanning from T7 to T9. Local adhesion was noted between the spinal cord and the tumor. Intraoperatively, a benign schwannoma was diagnosed through frozen section histology. Consequently, a T8 vertebrectomy with the removal of the intervertebral disc and the left pedicle of T7-T9 was performed. The tumor was meticulously excised, and bone grafting along with anterior fixation was implemented to ensure spinal stability.

**Figure 8:**
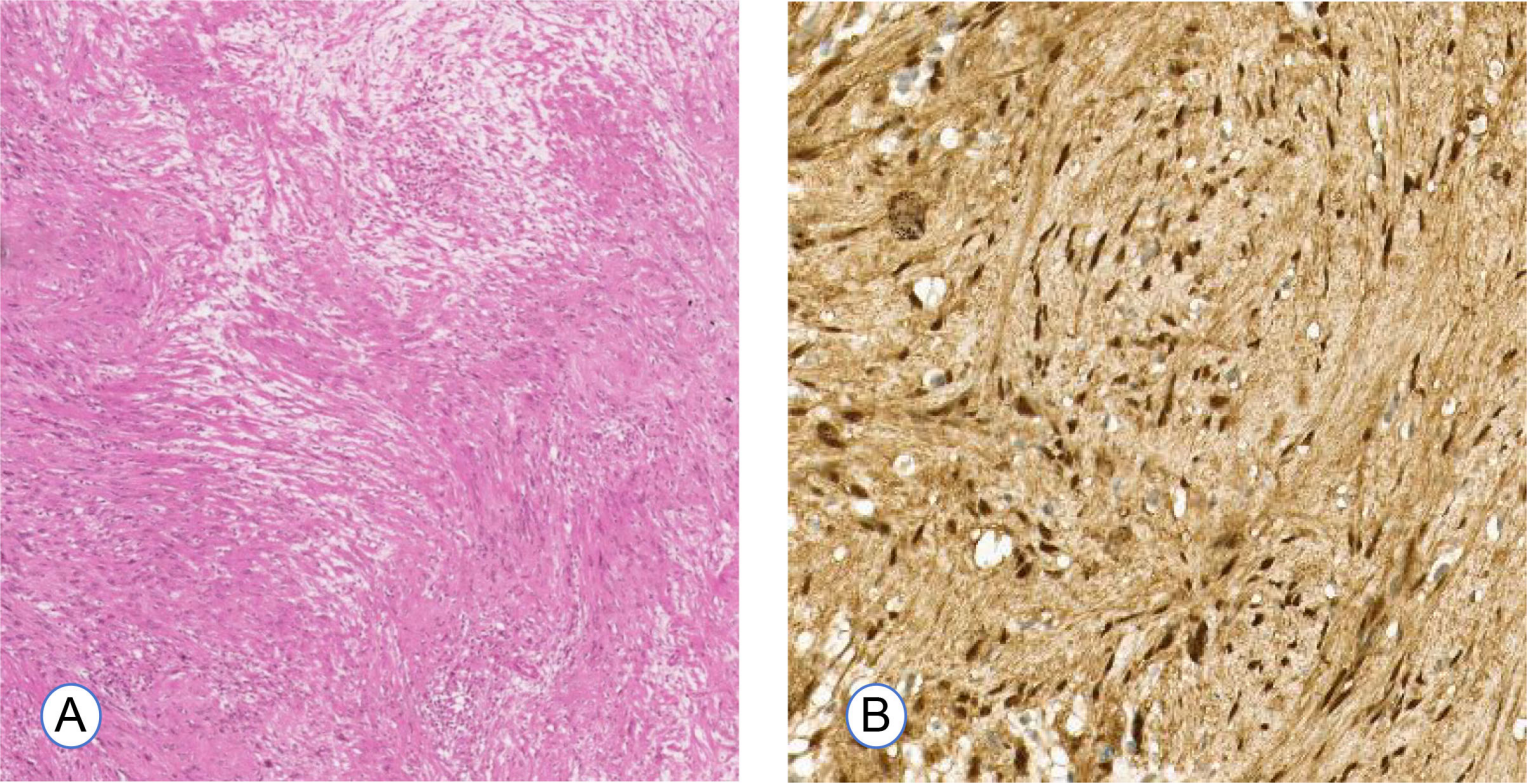
Histopathological examination of Case 2. **(A)** The photomicrograph of the tumor demonstrated hypocellular regions (Antoni A) and hypercellular regions (Antoni B) (H&E, original magnification ×4). **(B)** S-100 shows a diffuse positivity (brown color, ×10).

The histopathological examination revealed a characteristic spindle-cell architecture, featuring both Antoni A and B areas. Additionally, the immunohistochemical analysis indicated S-100 positivity and low Ki-67 proliferative activity (**Figure 8**). These histological findings corroborate the diagnosis of schwannoma.

**Figure 9:**
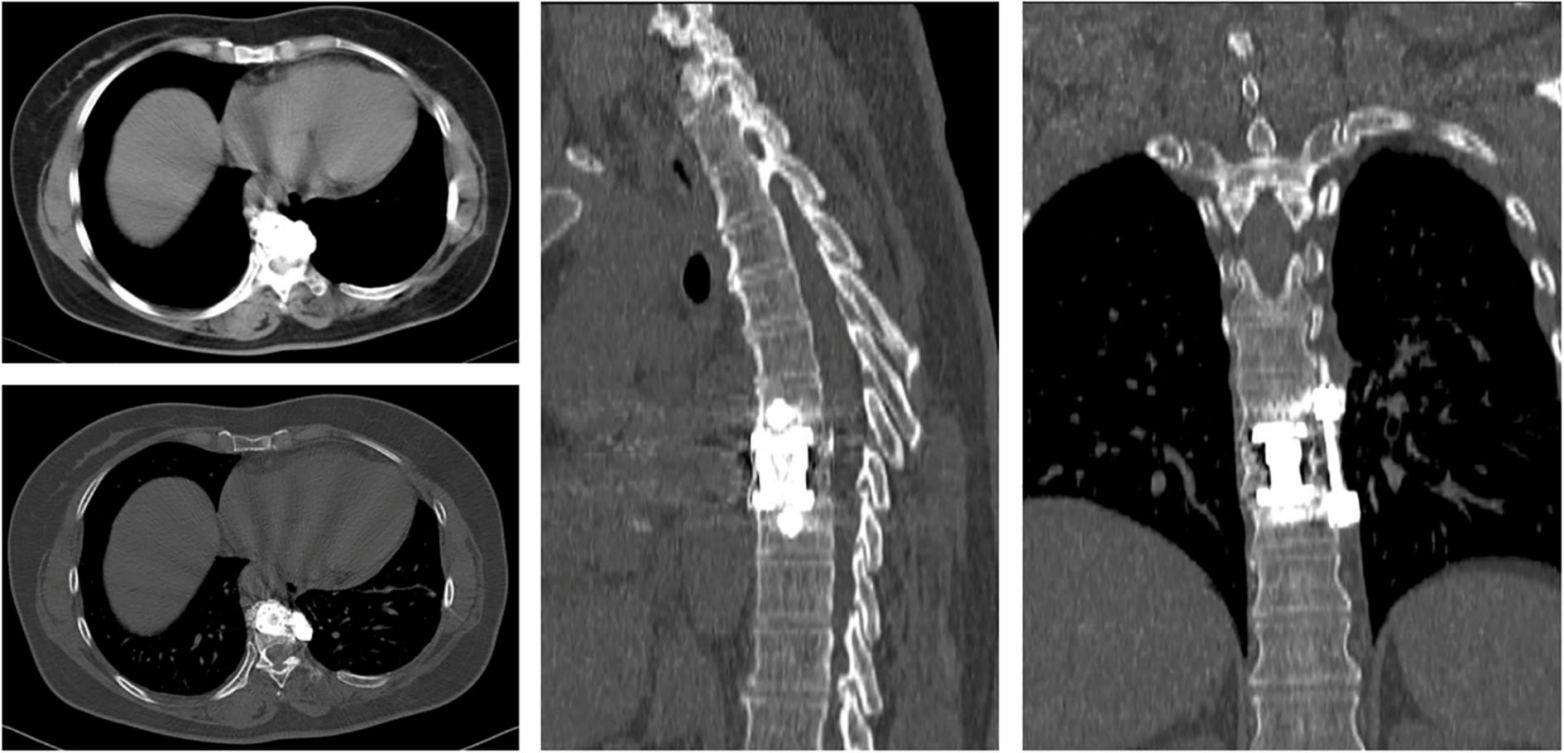
CT imaging reveals no evidence of recurrence during the 1-year follow-up period, with internal fixation in place.

At the 1-year follow-up, the patient exhibited no neurological deficits and showed no evidence of clinical or radiological recurrence (**Figure 9**).

## Discussion

Schwannomas are benign, well-circumscribed tumors of the peripheral nerve sheath, characterized by a clonal population of Schwann cells. These tumors can develop along any peripheral, cranial, or spinal nerve; however, they most frequently manifest as solitary tumors in the lumbar spine and the head and neck regions (5).

Intraosseous schwannomas (IOSs) are exceedingly rare, with spinal intraosseous schwannomas (SISs) representing an even more unusual subset. A review of 11 published cases of SISs located in the thoracic spine, including 2 from our institution (**Table 1**), revealed a broad age range among patients, with a predominance of middle-aged individuals [7/11] aged between 40 and 60 years, and no significant sex predilection observed. The most frequently involved level was T12 [4/11], followed by T9 [3/11]. Complete surgical excision was performed in the majority of cases [10/11], with spinal fusion conducted [8/11], predominantly via a posterior approach [6/11]. One immunocompromised patient underwent minimally invasive vertebroplasty due to the high risks associated with extensive tumor resection (6). All lesions [11/11] were pathologically benign, and no postoperative recurrences were reported.

Currently, there is no universally accepted definition or classification for SISs, which are crucial for determining the appropriate surgical approach. The PUTH classification encompasses all tumor types that can occur in the spine, dividing the sagittal axis into five sections (I to V) and the neural axis into four sections (A to D): I: posterior to the laminar; II: laminar/facet; III: intraspinal and posterior to the cord; IV: intraspinal and anterior to the cord; V: vertebral body; A: intraspinal and lateral to the cord; B: foraminal; C: extraforaminal (less than 4 cm away from the dural margin); D: far-extraforaminal (more than 4 cm away from the dural margin) (4). In this study, SIS is defined as a tumor that causes invasive and osteolytic destruction of the spine, regardless of the size of the extraosseous portion (PUTH classification Section V).

An enlarged foramen and scalloping of the vertebra result from pressure erosion as schwannomas increase in size. However, extensive destruction or erosion of the bone is uncommon. There are three possible mechanisms by which schwannomas can invade bone: (1) an extraosseous tumor causing secondary erosion of the bone; (2) a tumor originating in the nutrient canal and growing in a dumbbell configuration, leading to enlargement of the canal; (3) a tumor that arises primarily within the bone (7). Similar to most previous cases, the precise origin of our tumor could not be identified. One of our cases indicated that the majority of the tumor was intraosseous, and another presented as a paravertebral schwannoma originating from the T8 vertebral body. Consequently, the third mechanism mentioned above may represent the probable pattern of occurrence. Sherman reported that numerous nerve fibers exist within the marrow of the vertebra; however, most of these fibers appear to be non-myelinated (8). This observation explains why SISs arising from intraosseous nerve fibers are exceedingly rare (9).

The differentiation SISs from malignant spine tumors presents a significant diagnostic challenge due to overlapping features in clinical and imaging presentations. The clinical manifestations of SISs vary depending on the tumor’s size and location. Small SISs are typically asymptomatic; however, as the tumor enlarges, patients may experience localized pain or tenderness, neurological symptoms, weakness and numbness (10). Radiologically, SISs are characterized as expansile lytic lesions with sclerotic margins but without periosteal new bone formation, central calcification, or ossification. The size of SISs gradually increases, leading to foramen widening, vertebral scalloping, and paravertebral mass formation (11). On MRI imaging, typical schwannomas present as lobulated, encapsulated masses that appear hypointense relative to the spinal cord on T1WI and hyperintense on T2WI. Significant enhancement is noted following gadolinium administration (9). One of our cases demonstrates mild heterogeneous enhancement, which may be attributed to the absence of perfusion within the necrotic degeneration of the tumor. The imaging manifestations of SISs must be distinguished from a range of common osteolytic spinal lesions. These include chordoma, which typically occurs along the midline, presents as significantly high signal on T2-weighted images, and may show calcification; giant cell tumor of bone, which is commonly found in the young population and exhibits “soap bubble” expansive changes; aneurysmal bone cysts, which are prevalent in adolescents and characteristically display internal fluid-fluid levels and septal enhancement; metastatic spinal tumors, which usually have indistinct margins, are often multiple, and frequently have a history of primary malignancy; aggressive hemangiomas, which remain primarily osseous-centered and characteristically exhibit coarsened, thickened trabeculae on CT within the expanded vertebral body; and plasmacytomas or multiple myeloma, which usually present with multiple punched-out bone lesions.

Histological confirmation is essential for diagnosing SIS. Similar to schwannomas that develop in other locations, SIS exhibits a biphasic architecture, characterized by compact areas with nuclear palisading (Antoni A) and slightly looser areas containing microcystic regions (Antoni B) (12). Immunohistochemistry analysis of SIS reveals strong and diffuse nuclear and cytoplasmic positivity for the S-100 protein, which serves as a highly sensitive and characteristic marker (5). Additionally, these tumors consistently exhibit positivity for SOX10 (13). Staining for GFAP shows variability and may be positive in a subset of cases (14). Importantly, these tumors are negative for markers such as SMA, Desmin, EMA, Melan-A, and CD34, which aids in ruling out other spindle cell neoplasms. The Ki-67 proliferation index is characteristically low (<3%), further supporting its benign (WHO grade I) classification (15). The histopathological and immunohistochemical analyses of our two cases were consistent with the characteristics of schwannoma, and there was no evidence of malignancy.

Surgical excision is the preferred treatment for SIS. The surgical approach is typically determined by the tumor’s location, extent of involvement, and its anatomical relationship with surrounding critical structures. For anterior or anterolateral vertebral lesions with significant vertebral destruction, an anterior approach provides optimal exposure for resection and anterior column reconstruction. Conversely, tumors confined to the posterior elements or those extending into the posterior canal are best accessed via a posterior approach, which is more direct and less invasive. A combined anteroposterior approach is necessary when there is extensive involvement of both spinal columns or evidence of circumferential cord compression, allowing for complete resection, circumferential decompression, and 360° spinal stabilization. However, achieving radical complete resection can be challenging, particularly when paravertebral lesions invade nerve roots, the spinal cord, or surrounding major blood vessels. Given that SIS is benign, it is recommended to perform extensive curettage before utilizing a burr to remove the remaining lesion in the niches. Nonetheless, this surgical intervention often impacts spinal stability. To restore stability of the spine following lesion resection, bone grafting and internal fixation are advised. Immediate stability is reestablished using internal fixation devices, which create an optimal healing environment. Ultimately, through bone grafting, the bony healing process permanently eliminates unstable segments, thereby restoring the normal load-bearing function and anatomical alignment of the spine. In cases of gigantic SISs, the tumor capsule may have a rich blood supply; therefore, performing tumor angioembolization prior to surgery can help reduce and prevent significant intraoperative bleeding (16). In the surgical resection of our two patients, one was treated using an anterior approach alone, while the other underwent a combined anterior and posterior approach. Both patients received bone grafts and internal fusion to ensure spinal stability. Preoperative angioembolization was performed on one patient to minimize the risk of significant intraoperative bleeding.

In general, surgical excision or complete curettage are effective treatments for SISs, with recurrence being uncommon after surgical excision. Notably, only one case of recurrence involving malignant transformation of a benign SIS has been reported, occurring two years post-surgery (17). Throughout the follow-up period, no signs of recurrence were observed in our cases, and the patients remained asymptomatic.

## Conclusion

SIS should be included in the differential diagnosis of vertebral body osteolytic lesions, especially when characteristic imaging findings, such as neural foraminal widening and vertebral body scalloping, are present. On CT imaging, SISs present as osteolytic expansile lesions with sclerotic margins, lacking periosteal reaction or calcification. MRI reveals lobulated encapsulated masses that are iso- to hypointense on T1WI and hyperintense on T2WI, with homogeneous gadolinium enhancement, although necrotic areas may appear heterogeneous. These tumors may protrude into the spinal canal, leading to cord compression, or manifest as paraspinal soft tissue masses. However, a definitive diagnosis is contingent upon histopathological examination. The clinical symptoms and selection of surgical approaches associated with SIS vary depending on the tumor’s size and location. Complete tumor resection is the recommended treatment, and spinal fusion is often necessary for reconstruction, with recurrence being uncommon after surgery.

However, the absence of a universally accepted definition of SIS complicates the classification and management of these tumors. Therefore, further research is essential to achieve a clearer understanding and refined categorization of these complex tumors.

## Data Availability

The original contributions presented in the study are included in the article/supplementary material. Further inquiries can be directed to the corresponding authors.
